# Demographic and Social Characteristics of Internationally Educated Nurses in Sweden: Descriptive Statistical Study Comparisons Between Two Different Pathways for Recertification

**DOI:** 10.1177/23779608251313901

**Published:** 2025-02-26

**Authors:** Emina Hadziabdic, Kristofer Årestedt, Päivi Juuso, Anna-Maria Sarstrand Marekovic, Kristiina Heikkilä

**Affiliations:** 1 Department of Health and Caring Sciences, Faculty of Health and Life Sciences, 621093 Linnaeus University, Kalmar/Växjö, Sweden; 2 The Research Section, Region Kalmar County, Kalmar, Sweden; 3 Department of Health, Education and Technology, 91874 Luleå University of Technology, Luleå, Sweden; 4 Department of Social Studies, Faculty of Social Sciences, 621093 Linnaeus University, Växjö, Sweden

**Keywords:** cross-sectional survey, employment, immigration, internationally educated nurses, professional recertification, workforce integration

## Abstract

**Background:**

Nurse migration impacts global healthcare, which has a shortage of nurses, as many nurses move from lower-income to higher-income countries for better opportunities, working conditions, and salaries. Internationally educated nurses (IENs) have often been seen as a crucial solution to this issue. However, policies and regulations have been set in place to protect the public, including the recertification process and training to ensure educational comparability and competence. IENs’ contributions to the nursing workforce are significant, underscoring the importance of these policies and regulations.

**Aim:**

The aim was to describe the demographic and social characteristics of IENs who had completed recertification for nurses’ licenses in Sweden and to compare these characteristics among those who completed recertification through the National Board of Health and Welfare (NBHW) or bridging programs.

**Methods:**

A cross-sectional design using a survey and 818 questionnaires was sent to IENs with an identified postal address who had undergone the recertification process in Sweden. Of them, 296 (38%) were completed. Data were analyzed with descriptive statistics, chi-square tests, Fischer's exact tests, and independent sample t-tests.

**Results:**

Most IENs who had participated in a bridging program were women, commonly aged between 31 and 40 years of age who had immigrated mainly from Asian or Middle Eastern countries for family-related reasons. The average time to obtain a nursing license was 5.9 years, starting from the year they immigrated until recertification. IENs who received recertification by the NBHW were significantly younger (*p* < .001), had been in Sweden for a shorter time (*p* < .001), and the time to license was shorter (*p* < .001). Significant differences were also shown for origin (*p* < .001) and reason for immigration (*p* < .001).

**Conclusions:**

The findings can be used by decision-makers and authorities when developing higher education strategies for legalization and immigration policy to contribute to IENs’ career advancement opportunities.

## Introduction

The global nursing workforce was estimated to be 27.9 million in 2019 and 2020, reflecting the impact of ageing workforce patterns in healthcare and population growth (WHO, 2020). The COVID-19 pandemic exacerbated this shortage, necessitating rapid policy measures to increase the number of nurses (Buchan et al., 2022). However, increasing global migration has led to a significant increase in highly educated migrants like Internationally educated nurses (IENs) (Drennan & Ross, 2019; Mcaulife & Triandafyllidou, 2021). There are two main reasons for IEN migration: (1) voluntary migration related to personal reasons, such as family ties or improved quality of life, or professional factors, such as the search for work or improved working conditions and (2) forced migration due to war and/or persecution (Ng Chok et al., 2018).

Regardless of the cause of migration, IENs must enter the labor market as quickly as possible to integrate into society for the individual's sake so they can contribute to competence supply and multicultural diversity and offer their new skills and experience to society. As a result, many countries now need to focus on training new nurses to meet the rising demand (WHO, 2020). However, it has been challenging for IENs to obtain Swedish licensure. This is evidenced by the fact that between 2013 and 2018, the National Board of Health and Welfare (NBHW) in Sweden received approximately 2800 applications from IENs, of which only 346 were granted Swedish licensure (Ohlson, 2018). These challenges are also evident when IENs establish themselves in the labor market, as they face communication difficulties and barriers in the form of racism, discrimination, marginalization, mistreatment, fear of litigation and lawsuits, and differences in nursing practice even in countries with nursing shortages (Ghazal et al., 2020; Hopkins & Stephens, 2021).

## Review of Literature

Policies and regulations concerning IEN licensing to work in another country are critical for their integration into the labor market. Therefore, IENs are in need of support to integrate successfully into the healthcare settings of their new countries (Cubelo, 2024; Högstedt, 2024). IENs who want to become registered nurses must undergo a recertification process, including a licensing exam by a nursing regulatory body in the country where they plan to work. This is because nursing education and regulations differ between countries regarding what nurses are allowed to do, and there are differences in nursing education, national healthcare systems, nursing practice, and cultural values. The recertification aims to ensure the quality and safety of the nurses’ competencies and ensure that they meet the standards of the new country (Cubelo, 2024; Högstedt, 2024). Previous research has shown that recertification is lengthy, costly, and demanding (Covell et al., 2022; Ghazal et al., 2020).

In Sweden, the procedures for obtaining a nursing license for IENs have changed since 2007. However, a bridging program for IEN has existed in Sweden since the early 1990s. In 2015, the Swedish government initiated programs to find effective solutions to expedite the process for newly arrived immigrants to secure jobs in occupations experiencing workforce shortages, such as nursing. As a result, since 2016, there has been a significant expansion of the bridge program; from two to five universities that offer bridging programs. This expansion encompasses increased student participation in these programs and a broader range of higher education institutions offering bridging programs. European Union (EU) regulations have also affected the rules for people with educational backgrounds from countries outside the European Economic Area (EU/EEA) (Bengtsson & Viberg, 2019:3). For IENs, the process starts with an application to the NBHW to have their education assessed. The NBHW evaluates whether the education is likely comparable to the Swedish nursing program. If the NBHW determines that the education is sufficiently comparable, a decision is made to allow the nurse to proceed with the process through the board and undergo a recertification process through NBHW or a bridging program to obtain a Swedish nursing license.

Currently, there are two distinct pathways to recertification for nurses educated outside the EU/EEA in Sweden: (1) through the NBHW and (2) through the bridging program (see Table 1). For both pathways, the requirements for recertification are the same and can be found in the Higher Education Act (Swedish Council for Higher Education, 2016:157). The requirements include knowledge and skills equivalent to a Swedish nursing degree, knowledge of the Swedish constitution, and being capable of working in the Swedish language. The recertification process by the NBHW knowledge test is offered by one of the Swedish universities commissioned by the NBHW to oversee and conduct knowledge tests nationally for nurses trained outside the EU/EEA (Ohlson, 2018). The knowledge test consists of two parts: a written test and a practical test, meant to assess both theoretical and practical knowledge. The written test must be passed before IENs can register for the practical test. There are three opportunities per year for the written test and five opportunities per year for the practical test. Those who have passed the written test will receive a registration link for the practical test. The recertification process through the bridging program is available at five universities in Sweden, consists of 1 year of full-time studies (60 European Credit Transfer and Accumulkation System [ECTS] credits), and builds upon previous knowledge and experience. After successful completion of the bridge program, IENs may apply for Swedish licensure (Ohlson, 2018). The recertification process for IENs in Sweden is similar to that of other Nordic countries like Denmark, Finland, and Norway, but the implementation can vary. For example, Norway has a more simplified process for recognized foreign qualifications, while Finland provides more extensive integration programs to help IENs adapt to the local healthcare system (Cubelo et al., 2023; Nortvedt et al., 2024).

**Table 1.** The Process for IENs to Obtain a Swedish Nursing License.

Steps	Descriptions	Comments	
1	Educational assessment	An application is submitted to the NBHW to assess nursing education and determine its comparability to the Swedish nursing program. IEN is not required to know Swedish for this step	
2	Decision made by NBHW	The NBHW board authorized the IEN to either recertify through NBHW or complete a bridging program to obtain a Swedish nursing license	
3	Selection and fulfilling one of the pathways for recertification	NBHW recertification process	Bridging program recertification process
		The NBHW consist of the following: (1) complete the knowledge test that consists of a written test and a practical test (2) IEN needs to locate and fulfill 3 months of clinical placement (3) complete the course in laws and regulations for the Swedish health care sector 7,5 ECTS credits (4) attach a certificate of a language test to demonstrate IEN's proficiency in Swedish (Swedish at level C1 which indicates that the individual has an advanced language proficiency) when they apply for a license	The Bridging program consists of: (1) attach a certificate of a language test to demonstrate IEN's proficiency in Swedish (Swedish at level C1 which indicates that the individual has an advanced language proficiency) when they apply for a bridging program (2) complete 1 year of full-time studies (60 ECTS credits) following Swedish Law on Higher Education (2008:1101), which consists of theoretical and practical knowledge
4	Apply for a license	Once recertification is completed, IEN will confidently apply for a Swedish nursing license at NBHW.	

IEN= Internationally educated nurse; NBHW= the National Board of Health and Welfare.

Irrespective of the two different pathways for IEN recertification in Sweden (through NBHW or through the bridging program), IENs will only obtain a nursing occupational degree and become Licensed Practical Nurses (LPNs), instead of the two degrees—an academic (bachelor's) degree and an occupational (nursing) degree—which regular nursing students obtain. Nursing education in Europe is governed by EU directives (2005/36/EC, 2013/55/EU) as well as national legislation in each country. The nursing education program must comprise at least 180 ECTS credits, encompassing both theoretical and clinical training. According to the Swedish Higher Education ACT (2008:1101) and EU directives (2005/36/EC, 2013/55/EU), to obtain a bachelor's degree, IENs must complete further coursework on scientific methodology and compose a bachelor's thesis. The recertification process, whether it occurs through NBHW or via the bridging program, comprises a theoretical knowledge assessment of healthcare sector laws and regulations, as well as a clinical evaluation of the nursing skills required for practicing as registered nurses (RNs) in Swedish healthcare. The tests are held in Swedish (Bengtsson & Viberg, 2019:3). The pass rate of licensing exams through the recertification process by the NBHW is low, which has negative effects on both IENs and the Swedish healthcare system, which needs RNs (Montegrico, 2021). Therefore, the bridging program were developed to ease IENs’ recertification process and expand their cultural, practical, and theoretical knowledge to ensure patient safety (Covell et al., 2018; Cubelo et al., 2023; Hadziabdic et al., 2021; Högstedt et al., 2021; Nortvedt et al., 2020, 2024; Sherwood & Shaffer, 2014; Xu & He, 2012). These bridging programs have been effective: 79% of nurses who completed the bridging programs in Sweden held positions in the labor market after 1 year (Bengtsson & Viberg, 2019, p. 3).

A single previous comparative study on the demographic and social characteristics of IENs who had completed recertification for nurses’ licenses exposed that IENs who arrived after 2002 possessed high levels of knowledge, professional experience, and language proficiency. Participation in bridging programs and support from social networks in Canada significantly predicted employment challenges for IENs (Covell et al., 2017). Previous qualitative studies of IENs’ experiences of bridging programs showed that they facilitated IENs’ integration into the nursing profession (Covell et al., 2018; Hadziabdic et al., 2021; Högstedt, 2024; Högstedt et al., 2021; Nortvedt et al., 2024). However, IENs have experienced a lack of information and knowledge about the recertification procedures, a lack of transparency in assessing requirements and language proficiency, and they have found the recertification process to be costly and stressed that the process of comparing previous educational and professional qualifications was unclear (Cubelo et al., 2023; Hadziabdic et al., 2021). IENs have also reported that bridging programs should address culturally patterned responses among different groups of IENs (Brunton et al., 2019). Moreover, Scandinavian research has highlighted the risk that the recertification process may lead to deprofessionalization (deskilling) for IENs, due to a mismatch of expectations between domestically trained nurses and IENs concerning the scope of practice and nursing (Korzeniewska & Erdal, 2021; Nortvedt et al., 2024). Given the global nursing shortage, there is an increasing demand for IENs in many countries. Consequently, there is a pressing need to speed up nursing education (Buchan et al., 2022; WHO, 2020). Moreover, insight into the characteristics of those seeking recertification is needed to streamline the recertification process.

A comprehensive understanding of the various characteristics of IENs who have completed recertification by different paths is crucial in facilitating their integration into the labor market. Additionally, conducting a comparative analysis of these characteristics among those who have completed recertification through NBHW or through a bridging program can offer valuable insights and skills that employers can leverage. This is particularly important given the growing diversity in society, and can help to facilitate the seamless integration of IENs into the workforce. The aim of the study was to describe the demographic and social characteristics of IENs who had completed recertification for nurses’ licenses in Sweden and to compare these characteristics among those who completed the recertification through NBHW or through a bridging program.

## Methods

### Design

This was a cross-sectional descriptive study (Polit & Beck, 2022) and involved distributing 818 self-administered questionnaires to IENs. The work adhered to STROBE guidelines for cross-sectional studies (Cuschieri, 2019).

### Inclusion and Exclusion Criteria

The present study included IENs from outside the EU/EES who had undergone the recertification process, which resulted in their acquisition of a registered nursing license in Sweden from January 2012 to June 2022.

### Sample and Recruitment

IENs were identified through the nursing regulatory body (NBHW in Sweden) which sent out the names of all IENs who had received nursing licenses between January 2012 and June 2022 (n = 830). The contact information and addresses for the IENs were acquired through a manual search of the official contact internet webpages (www.hitta.se and www.eniro.se). These official websites list all residents in Sweden by law unless they have hidden identities. An individual who does not have a hidden identity and wants to be excluded from official websites must take the initiative to proactively remove all their personal information from such platforms. All addresses except 12 were identified.

Written information about the study, along with a questionnaire and a prepaid envelope was posted to the identified IENs with an identified postal address (n = 818), to be returned to the principal investigator. The research team's contact details were included in case the participants had any questions. In total, 40 postcards were returned due to wrong address. One reminder was posted to all 778 IENs after 4 weeks, since the study collected data anonymously.

**Figure 1.**
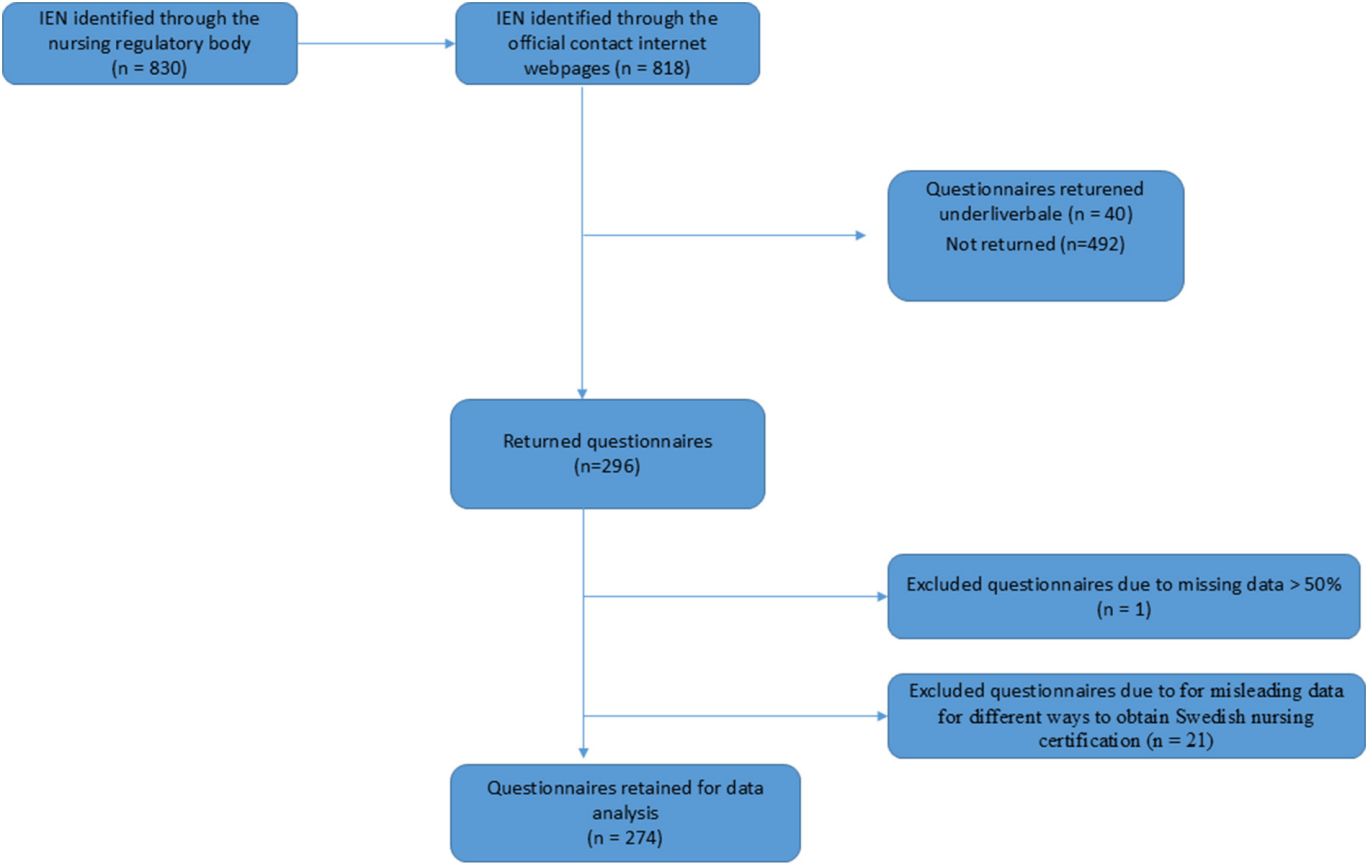
Sampling frame.

In total, 296 questionnaires were returned, giving a response rate of 38%. Twenty-two of the questionnaires had incomplete responses, and one questionnaire missing more than 50% of its data was excluded (Figure 1). Furthermore, 21 of the questionnaires provided contradictory responses for three variables of main interest pertaining to the different ways of obtaining Swedish nursing certifications. As it was not possible to classify them into the NBHW or to bridging program group, these questionnaires were also excluded. Consequently, the study sample consisted of 274 IENs.

### Data Collection

A 24-item study-specific self-administered questionnaire was developed based on a previous qualitative study concerning IENs’ experiences with a Swedish bridging program and the program's role in their integration into the nursing profession (Hadziabdic et al., 2021). When formulating questions, the results from the previous qualitative study were organized into three different sections. The first section included six demographic questions (gender, age, cause of migration, year of residence in Sweden, country of birth, and mother language). The second section included seven questions about the IEN's personal and social situation upon immigration; for example, education level, education country, and working experience. The third and final section included eight questions about the social situation after immigration; for example, working experience after immigration before they received their Swedish nursing license, ways to obtain a Swedish nursing certification, current working situation, and the process of finding a job as a registered nurse. The questionnaire was written in Swedish and included three open-ended questions, which allowed IENs to write comments on their answers in their own words. These open-ended questions have not been used in the present study. To ensure content validity, the questionnaire was discussed by experienced researchers with expert knowledge in quantitative and migration studies and then pilot-tested in ten IENs. This resulted in minor changes in the wording (Polit & Beck, 2022).

### Ethical Approval and Informed Consent Statements

The Ethical Review Authority approved the study (No. 2021-03844). The participants were reached through postal mail and were provided with a questionnaire and written details about the study's purpose, their freedom to refuse or discontinue participation at any time, and contact information for the project team. Completion and submission of the questionnaire indicated consent. To ensure anonymity, all names were substituted with ID numbers upon survey completion (World Medical Association, 2013).

### Statistical Analysis

Descriptive statistics were used to present the sample and study variables; categorical data (i.e., nominal and ordinal variables, range, and n (%)) were described with frequencies and percentages, while continuous data were described with means and standard deviations. To compare the two different pathways to IEN recertification—that is, NBHW versus bridging program—chi-squared test or Fisher's exact test was used for categorical data and independent sample t-test was used for continuous data. Statistical significance was defined as *p* < .05. Analyses were conducted in SPSS version 29 for Windows (SPSS Inc, Chicago, IL, USA).

## Results

### Sample Characteristics and Research Question Results

The mean age of the IENs was 41.8 (SD = 8.4; range = 27–70) years, and the majority were females (n = 230; 83.9%). More than half originated from Asia (n = 104; 38.1%) or from Middle Eastern countries (n = 57, 20.9%) and stated that their reason for migration to Sweden was family-related (n = 156; 56.9%). Only a small number of respondents stated that they had migrated to Sweden involuntarily as refugees (n = 35; 12.8%). More than half of IENs had immigrated to Sweden during the past 10 years (n = 156; 57.1%). A great majority of IENs had worked as assistant nurses (n = 204; 74.5%) before the recertification, followed by teachers (n = 11; 4.0%), waitresses or waiters (n = 4; 1.5%), cleaners (n = 2; 0.7%), or other profession (n = 6; 2.2%; Table 2).

**Table 2.** Demographic and Social Characteristics of the IENs in Relation to Pathways for Recertification.

		Pathways for recertification		
	All, n = 274	National Board of Health and Welfare, n = 89	Bridging program, n = 185	*p*-Value
Age (years), mean (SD) [range]	41.8 (8.4) [24–70]	38.7 (7.8) [24–65]	43.2 (8.3) [26–70]	<.001^a^
Age (years grouped), n (%)				.001^b^
≤30	18 (6.6)	12 (13.5)	6 (3.3)	
31–40	112 (41.0)	43 (48.3)	69 (37.5)	
41–50	97 (35.5)	26 (29.2)	71 (38.6)	
≥51	46 (16.9)	8 (9.0)	38 (20.7)	
Missing	1	–	1	
Sex, n (%)				.839^b^
Female	230 (83.9)	73 (82.0)	157 (84.9)	
Male	42 (15.3)	14 (15.7)	28 (15.1)	
Other	2 (0.7)	2 (2.3)	0 (0.0)	
Born, n (%)				<.001^c^
Middle East	57 (20.9)	21 (23.6)	36 (19.6)	
Africa	38 (13.9)	2 (2.3)	36 (19.6)	
Asia	104 (38.1)	35 (39.3)	69 (37.5)	
South America	11 (4.0)	4 (4.5)	7 (3.8)	
North America	12 (4.4)	9 (10.1)	3 (1.6)	
Australia	4 (1.5)	4 (4.5)	–	
Europe	38 (13.9)	7 (7.9)	31 (16.9)	
Sweden	9 (3.3)	7 (7.9)	2 (1.1)	
Missing	1	–	1	
Reason for migration, n (%)				<.001^b^
Family related	156 (56.9)	30 (33.7)	126 (68.1)	
Work related	42 (15.3)	29 (32.6)	13 (7.0)	
Refugee	35 (12.8)	6 (6.7)	29 (15.7)	
Studies	17 (6.2)	9 (10.1)	8 (4.3)	
Other	24 (8.8)	15 (16.9)	9 (4.9)	
Years in Sweden, mean (SD) [range]	10.1 (5.4) [1–33]	6.8 (4.4) [1–31]	11.7 (5.2) [2–33]	<.001^a^
Years in Sweden (grouped), n (%)				<.001^b^
≤10	156 (57.1)	71 (80.7)	85 (46.0)	
11–20	100 (36.6)	14 (15.9)	86 (46.5)	
≥21	17 (6.2)	3 (3.4)	14 (7.6)	
Missing	1	1	–	
Professional work experience in Sweden before license validation, n (%)				.149^c^
Assistant nurse	204 (74.5)	65 (87)	139 (90.8)	
Cleaner	2 (0.7)	–	2 (1.3)	
Teacher	11 (4.0)	5 (6.8)	6 (3.9)	
Waitress/waiter	4 (1.5)	–	4 (2.8)	
Other	6 (2.2)	4 (5.4)	2 (1.3)	
Missing	47	15	32	
Time license (years), mean (SD) [range]	5.9 (4.3) [<1–29]	3.8 (3.6) [<1–29]	6.9 (4.3) [<1–26]	<.001^a^
Professional work experience as a nurse after receiving the Swedish nurse license, n (%)				.085^c^
Primary healthcare	17 (6.3)	5 (6.3)	12 (6.7)	
Outpatient clinics	26 (9.7)	7 (8.0)	19 (10.6)	
Specialized care at the hospital	15 (5.6)	5 (5.7)	10 (5.6)	
Hospital care award	132 (49.3)	35 (39.8)	97 (53.9)	
Community care	71 (24.1)	32 (36.4)	39 (21.7)	
Other care context	7 (2.4)	4 (4.5)	3 (1.7)	
Missing	6	1	5	

^a^ Independent sample t-tests.

^b^ Pearson’s chi-square test.

^c^ Fisher's exact test.

IEN= Internationally educated nurse.

About two-thirds of IENs reported that they had taken part in a bridging program (n = 185; 67.5%) and one-third (n = 89; 32.5%) had gone through the recertification process by the NBHW. The time for recertification was on average 5.9 (SD = 4.3) years, and once the IENs had successfully obtained their nursing license, a majority went on to work as registered nurses in hospital care ward settings (n = 132; 49.3%) or community care (n = 71; 24.1%; Table 2).

Statistically significant differences were shown between the two pathways for recertification regarding age, origin, reason for immigration, years of residence in Sweden, and time to nursing license (Table 2).

IENs who successfully completed recertification through the NBHW were significantly younger (*p* < .001) and had been in Sweden for a shorter period of time (*p* < .001) compared to IENs who completed the recertification through a bridging program (Table 2).

National origin differed significantly between the two groups (*p* < .001); IENs who completed recertification through an NBHW were more often from Asia, the Middle East, and North America, while IENs who completed the recertification through a bridging program were more often from Asia, Middle East, Africa, and Europe (Table 2).

A significant difference was also shown regarding the reason for migration (*p* < .001); work- and study-related reasons were more common in IENs who completed recertification through NBHW, while family-related reasons and refugee status were more common among IENs who completed their recertification through a bridging program (Table 2).

Finally, the time to license was significantly shorter for IENs who completed the recertification through the NBHW compared to through a bridging program (*p* < .001; Table 2).

## Discussion

This survey study is, to our best knowledge, the first that has profiled the characteristics of IENs applying for recertification to be registered nurses in Sweden. Furthermore, it is the first study to compare the characteristics of those who validated their license through the NBHW and through the bridging program. The study thus contributes relevant knowledge on IENs and their pathways to becoming RNs in Sweden.

The study found that IENs were mostly female, middle-aged, and had immigrated due to family-related reasons over the past 10 years. The vast majority of IENs in our study were born in Asia and had chosen to pursue recertification by enrolling in a bridging program, rather than seeking recertification through the NBHW. It is worth noting that disparities were observed among the IENs who acquired their nursing license through recertification through the NBHW compared to those who completed the bridging program. IENs who obtained the nursing license through recertification through the NBHW tended to be younger and had immigrated to Sweden due to work-related reasons. They also had a shorter duration of stay in Sweden before acquiring their nursing license compared to those who participated in a bridging program.

The study revealed that IENs were mostly born in Asia, were female, and had immigrated voluntarily; for example for family- or work-related reasons. Furthermore, they lived in Sweden for nearly 6 years before obtaining their nursing license. This finding reflects contemporary migration patterns in Sweden in recent years (Statistics of Sweden, 2023), in that it takes a longer time for immigrant women to become established in working life due to ill health, family situations, parental leave, housing situations, waiting for childcare, and starting school later than men (Cheung et al., 2017). Additionally, the findings indicate that Asia is the largest region of origin for IENs. This is particularly noteworthy given that Sweden does not have an active recruitment strategy to address nursing shortages, unlike Finland, where IENs are predominantly recruited from Asia—especially the Philippines (Cubelo, 2023a). The findings point out that IENs’ human capital, social capital, and health status both during and after migration play a significant role in the employment process (Hadziabdic et al., 2021; Högstedt et al., 2021). This can be attributed to employers being aware of the fact that the IEN group is a heterogeneous group with various types of training, healthcare systems, and preparation but with a common denominator of self-efficiency (Högstedt et al., 2022), enabling IENs’ entry into the nursing workforce.

This study found that most IENs acquired professional recertification through participation in bridging programs, in contrast to a previous study by Covell et al. (2017) which found that most IENs did not participate in bridging programs. The higher participation rate in the bridging program may be due to several reasons. One of these reasons could be related to changes in Swedish higher education legalization concerning education from countries outside the EU/EEA (2016, p. 157) or to a lack of RNs in Swedish healthcare (Regulation on the Recognition of Professional Qualifications, 2016:157). This law was established to create a legally secure and effective system for the recertification of healthcare education from outside the EU/EEA and to cover the entire recertification process from the application for assessment of education outside the EU/EEA up to the issuance of identification (Bengtsson & Viberg, 2019:3). Furthermore, one task has been to identify and delimit the division of responsibilities among the NBHW, the Swedish National Agency for Higher Education, universities and colleges, county councils, and labor market authorities in the various parts of the recognition process. The implementation of this law was intended to reduce the time required to gain qualification and thus facilitate access to the Swedish labor market. As a result, bridging programs were established to facilitate a smoother transition for IENs into Swedish nursing practice. Starting in 2016, bridging programs have significantly expanded, both in terms of the number of available programs and the increased funding allocated to universities. Specifically, funding has grown from SEK 51 million in 2012 to SEK 202 million in 2018. Consequently, the greater availability of bridging programs has been distributed across urban and semiurban centers, enabling participation in the program throughout the country (Bengtsson & Viberg, 2019, p. 3).

This study found that a significant proportion of IENs have opted for the bridging program to pursue recertification instead of seeking recertification through the NBHW. It is worth noting that many of these IENs had resided for longer in Sweden and were older when they obtained their nursing license before obtaining it compared to those who validated the nursing license through the NBHW. A previous study has indicated that age influences successful workplace integration for IENs, with younger individuals demonstrating higher language proficiency and a greater likelihood of success than their older counterparts (Guven & Islam, 2015). Additionally, female nurses over the age of 45 face an increased risk of gender- and age-related discrimination (Kagan & Melendez-Torres, 2015). Given these factors, it is crucial for policymakers and employers to consider these insights when developing support strategies and employment-related policies for the work-life transition of IENs who obtained their nursing licenses through bridging programs.

The present study reveals that prior to the recertification process, IENs predominantly worked as nursing assistants. Previous research indicates that Filipino nurses who migrated to Norway often ended up in nursing assistant roles due to difficulties in obtaining a nursing license from healthcare authorities, which led to deskilling (Nortvedt et al., 2020). Further, previous studies in Finland and Norway found that the lack of recognition of nursing education and qualifications from outside the EU/EEA by national health authorities results in deskilling and prevents IENs from practicing their profession in their new countries (Cubelo, 2023b; Nortvedt et al., 2024). The finding also highlights the importance of policymakers in developing recertification approaches to assist IENs in obtaining a bachelor's degree in nursing, which would contribute to career advancement opportunities (Cubelo, 2023b; Korzeniewska & Erdal, 2021; Nortvedt et al., 2024).

Another reason for the high proportion of participation in bridging programs can be related to the participant's reasons for migrating. The participants in the bridging program reported that a higher proportion were refugees, and this could be interpreted as meaning they were more in need of formal assistance, such as bridging programs, to become familiar with the Swedish healthcare system, the nurse's role, and clinical placement (Covell et al., 2018; Hadziabdic et al., 2021; Högstedt et al., 2021). Recognizing social factors such as country of origin, years in the host country, previous learning and experience of IENs, and migration reasons can help bridging programs better support IENs in enhancing their professional skills. This strategy also promotes diversity and resilience within the healthcare workforce (Cubelo, 2024; Cubelo et al., 2024). The third observed association may be attributed to not all bridging programs requiring proficiency exams, including theoretical and practical examinations, but all of them incorporate clinical placements. Furthermore, a significant proportion of this program is concentrated in metropolitan regions, where many immigrants reside. This geographical distribution could also account for the high participation rates in this program.

### Limitations

For the present study, a study-specific questionnaire was developed, which was based on a previous qualitative study (Hadziabdic et al., 2021). To ensure content validity, the questionnaire was pilot-tested and discussed by experienced researchers with expert knowledge in quantitative and migration studies.

Another limitation of the study is its low response rate, which may affect the external validity and, thus, limit the generalizability of the results. It is known that the response rate, in general, is lower among immigrants, particularly immigrants with a non-Western background than among domestic populations. A low response rate among immigrants could be explained due to alienation generated by the questions’ focus on cultural assumptions, and mistrust regarding anonymity (Ahlmark et al., 2015). Measures were taken to increase survey participation; for example, a cover letter containing the name of the sender and the logo of the research institute and providing information about the purpose and anonymity of the study was sent to the participant's homes (Ahlmark et al., 2015).

Finally, since the present study had a cross-sectional design, no causal relationships can be established (Polit & Beck, 2022).

### Implications for Practice

The study contributes to emerging evidence of the demographic, educational, and professional resources and strategies that may influence IENs’ ability to obtain a registered nursing license and contribute to workforce integration. The findings can provide input for decision-makers and authorities when developing strategies in higher education legalization concerning countries outside the EU/EES to contribute to IENs’ career advancement opportunities and to integrate them into healthcare workforces.

## Conclusion

The findings showed differences in age, origin, the reason for immigration, duration of stay, and the time to license in Sweden in the group of IENs who had received recertification through the NBHW and those who had done so through the bridging program. IENs who acquired their nursing licenses through recertification with the NBHW exhibited demographic characteristics that included a younger age profile, originated more often from Asia, the Middle East, and North America, and tended to immigrate to Sweden for work-related purposes. Additionally, this group demonstrated a shorter duration of residency in Sweden before obtaining their nursing licenses compared to those who engaged in bridging programs. Irrespective of the heterogeneity in the group of IENs and the process they chose for recertification, this study's findings show that most of them worked as nurses after receiving the Swedish nurse license. IENs, often with previous experience from work in healthcare, are a valuable resource in the capacity-building of the nursing workforce. Shortening the time required to obtain a license is essential, both for the IENs and the receiving societies. These findings can inform decision-makers and authorities in developing higher education strategies, legalization processes and immigration policies to support IENs’ workforce integration and career advancement.

## Supplemental Material

sj-jpg-1-son-10.1177_23779608251313901 - Supplemental material for Demographic and Social Characteristics of Internationally Educated Nurses in Sweden: Descriptive Statistical Study Comparisons Between Two Different Pathways for Recertification Supplemental material, sj-jpg-1-son-10.1177_23779608251313901 for Demographic and Social Characteristics of Internationally Educated Nurses in Sweden: Descriptive Statistical Study Comparisons Between Two Different Pathways for Recertification by Emina Hadziabdic, Kristofer Årestedt, Päivi Juuso, Anna-Maria Sarstrand Marekovic and Kristiina Heikkilä in SAGE Open Nursing

sj-jpg-2-son-10.1177_23779608251313901 - Supplemental material for Demographic and Social Characteristics of Internationally Educated Nurses in Sweden: Descriptive Statistical Study Comparisons Between Two Different Pathways for Recertification Supplemental material, sj-jpg-2-son-10.1177_23779608251313901 for Demographic and Social Characteristics of Internationally Educated Nurses in Sweden: Descriptive Statistical Study Comparisons Between Two Different Pathways for Recertification by Emina Hadziabdic, Kristofer Årestedt, Päivi Juuso, Anna-Maria Sarstrand Marekovic and Kristiina Heikkilä in SAGE Open Nursing

sj-jpg-3-son-10.1177_23779608251313901 - Supplemental material for Demographic and Social Characteristics of Internationally Educated Nurses in Sweden: Descriptive Statistical Study Comparisons Between Two Different Pathways for Recertification Supplemental material, sj-jpg-3-son-10.1177_23779608251313901 for Demographic and Social Characteristics of Internationally Educated Nurses in Sweden: Descriptive Statistical Study Comparisons Between Two Different Pathways for Recertification by Emina Hadziabdic, Kristofer Årestedt, Päivi Juuso, Anna-Maria Sarstrand Marekovic and Kristiina Heikkilä in SAGE Open Nursing

sj-jpg-4-son-10.1177_23779608251313901 - Supplemental material for Demographic and Social Characteristics of Internationally Educated Nurses in Sweden: Descriptive Statistical Study Comparisons Between Two Different Pathways for Recertification Supplemental material, sj-jpg-4-son-10.1177_23779608251313901 for Demographic and Social Characteristics of Internationally Educated Nurses in Sweden: Descriptive Statistical Study Comparisons Between Two Different Pathways for Recertification by Emina Hadziabdic, Kristofer Årestedt, Päivi Juuso, Anna-Maria Sarstrand Marekovic and Kristiina Heikkilä in SAGE Open Nursing

sj-jpg-5-son-10.1177_23779608251313901 - Supplemental material for Demographic and Social Characteristics of Internationally Educated Nurses in Sweden: Descriptive Statistical Study Comparisons Between Two Different Pathways for Recertification Supplemental material, sj-jpg-5-son-10.1177_23779608251313901 for Demographic and Social Characteristics of Internationally Educated Nurses in Sweden: Descriptive Statistical Study Comparisons Between Two Different Pathways for Recertification by Emina Hadziabdic, Kristofer Årestedt, Päivi Juuso, Anna-Maria Sarstrand Marekovic and Kristiina Heikkilä in SAGE Open Nursing

sj-jpg-6-son-10.1177_23779608251313901 - Supplemental material for Demographic and Social Characteristics of Internationally Educated Nurses in Sweden: Descriptive Statistical Study Comparisons Between Two Different Pathways for Recertification Supplemental material, sj-jpg-6-son-10.1177_23779608251313901 for Demographic and Social Characteristics of Internationally Educated Nurses in Sweden: Descriptive Statistical Study Comparisons Between Two Different Pathways for Recertification by Emina Hadziabdic, Kristofer Årestedt, Päivi Juuso, Anna-Maria Sarstrand Marekovic and Kristiina Heikkilä in SAGE Open Nursing

sj-jpg-7-son-10.1177_23779608251313901 - Supplemental material for Demographic and Social Characteristics of Internationally Educated Nurses in Sweden: Descriptive Statistical Study Comparisons Between Two Different Pathways for Recertification Supplemental material, sj-jpg-7-son-10.1177_23779608251313901 for Demographic and Social Characteristics of Internationally Educated Nurses in Sweden: Descriptive Statistical Study Comparisons Between Two Different Pathways for Recertification by Emina Hadziabdic, Kristofer Årestedt, Päivi Juuso, Anna-Maria Sarstrand Marekovic and Kristiina Heikkilä in SAGE Open Nursing

sj-docx-8-son-10.1177_23779608251313901 - Supplemental material for Demographic and Social Characteristics of Internationally Educated Nurses in Sweden: Descriptive Statistical Study Comparisons Between Two Different Pathways for Recertification Supplemental material, sj-docx-8-son-10.1177_23779608251313901 for Demographic and Social Characteristics of Internationally Educated Nurses in Sweden: Descriptive Statistical Study Comparisons Between Two Different Pathways for Recertification by Emina Hadziabdic, Kristofer Årestedt, Päivi Juuso, Anna-Maria Sarstrand Marekovic and Kristiina Heikkilä in SAGE Open Nursing
