# Supplementary figures and images for: Demographic and Social Characteristics of Internationally Educated Nurses in Sweden: Descriptive Statistical Study Comparisons Between Two Different Pathways for Recertification
Source: SAGE Open Nurs. 2025 Feb 26;11:23779608251313901. doi: 10.1177/23779608251313901 (PMC11866370; doi:10.1177/23779608251313901)

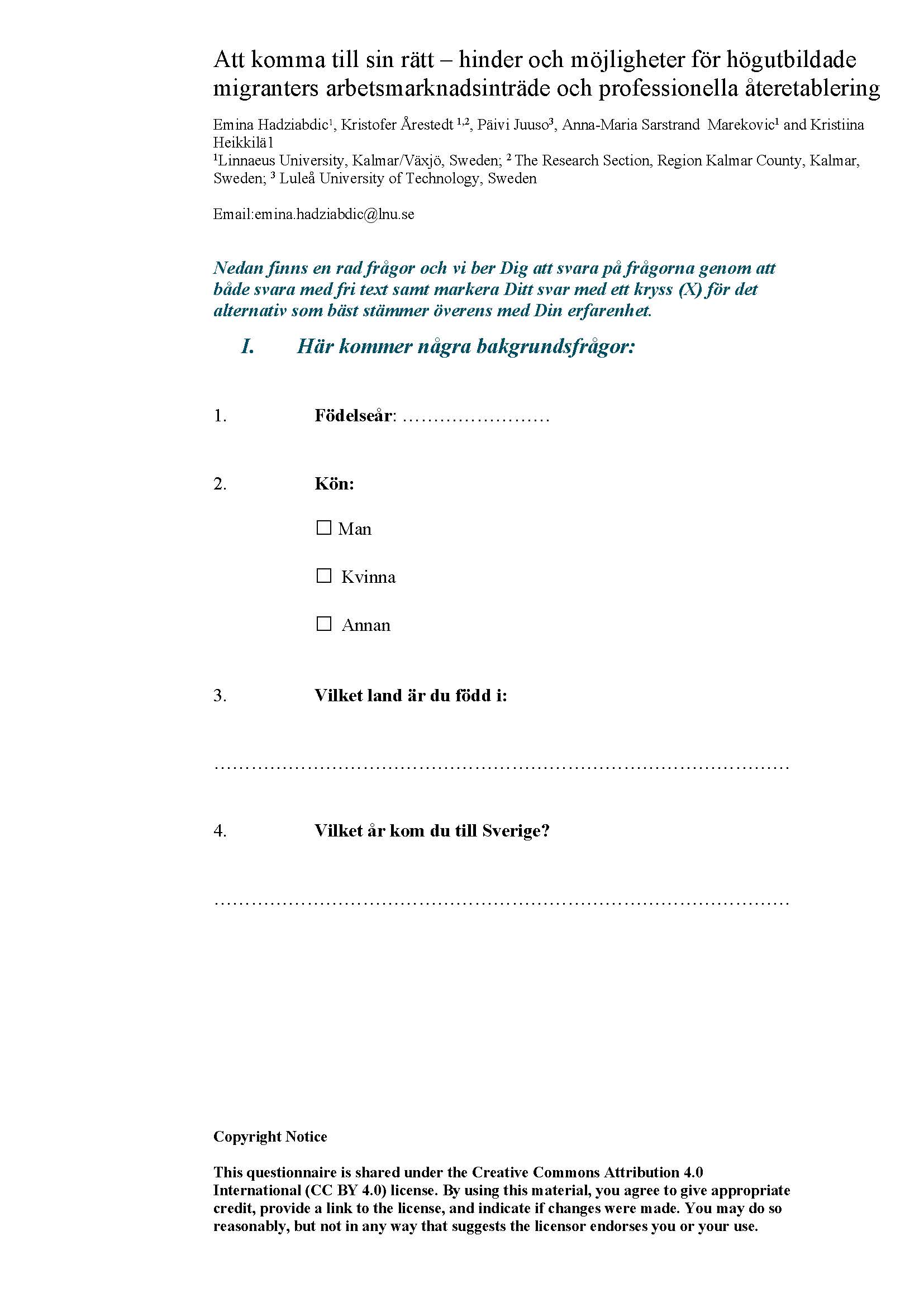

Supplement: sj-jpg-1-son-10.1177_23779608251313901 - Supplemental material for Demographic and Social Characteristics of Internationally Educated Nurses in Sweden: Descriptive Statistical Study Comparisons Between Two Different Pathways for Recertification [file sj-jpg-1-son-10.1177_23779608251313901.jpg]

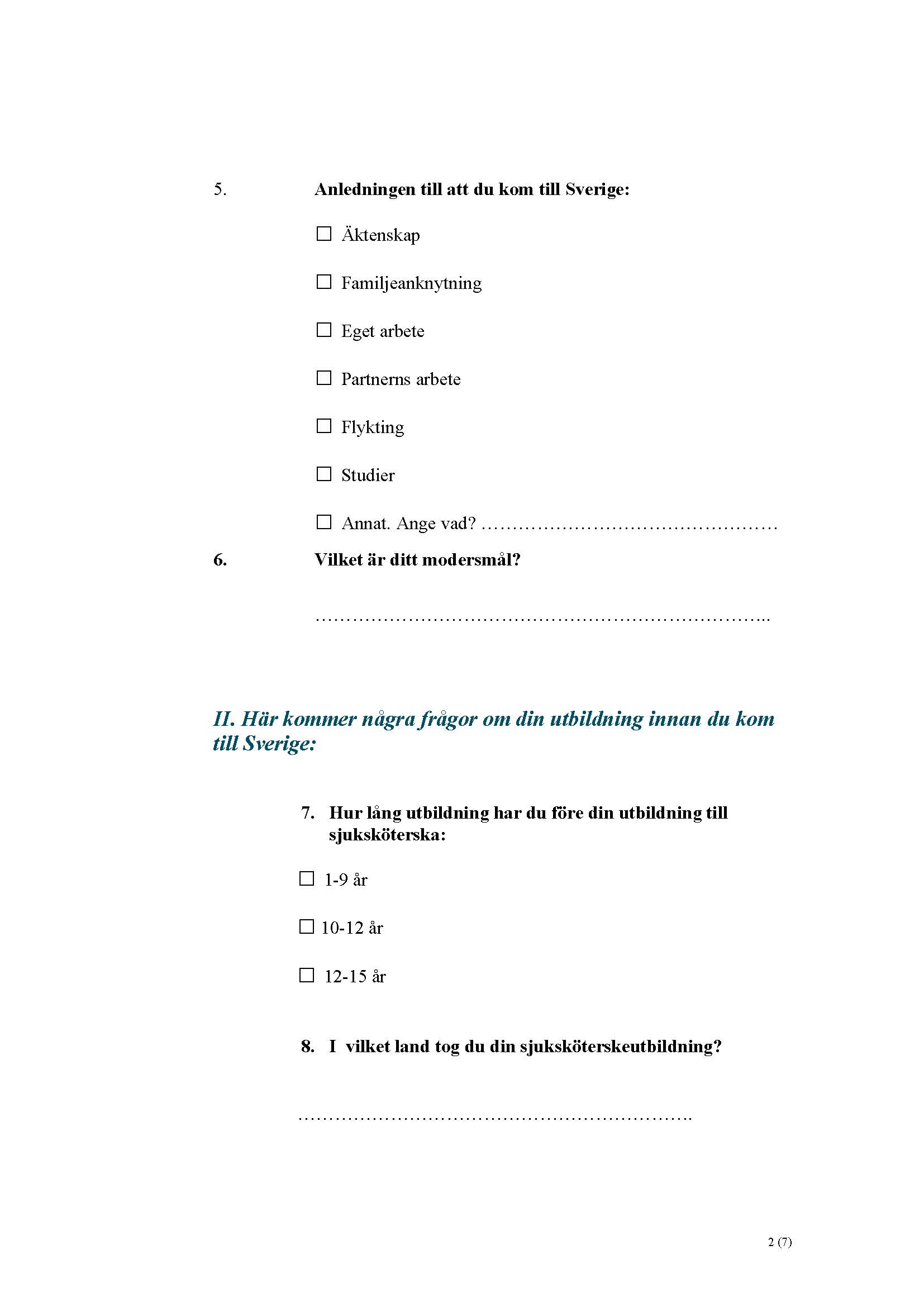

Supplement: sj-jpg-2-son-10.1177_23779608251313901 - Supplemental material for Demographic and Social Characteristics of Internationally Educated Nurses in Sweden: Descriptive Statistical Study Comparisons Between Two Different Pathways for Recertification [file sj-jpg-2-son-10.1177_23779608251313901.jpg]

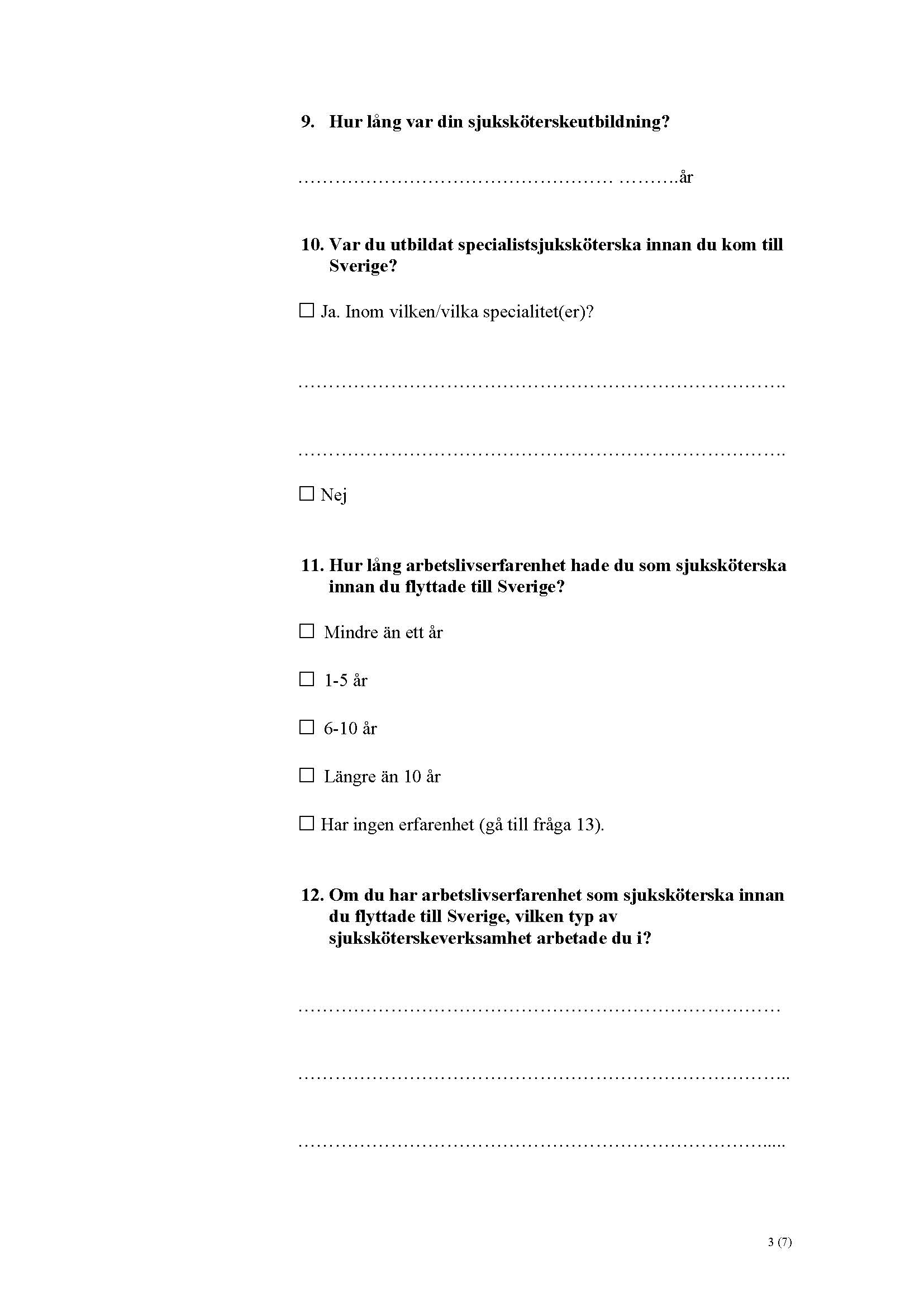

Supplement: sj-jpg-3-son-10.1177_23779608251313901 - Supplemental material for Demographic and Social Characteristics of Internationally Educated Nurses in Sweden: Descriptive Statistical Study Comparisons Between Two Different Pathways for Recertification [file sj-jpg-3-son-10.1177_23779608251313901.jpg]

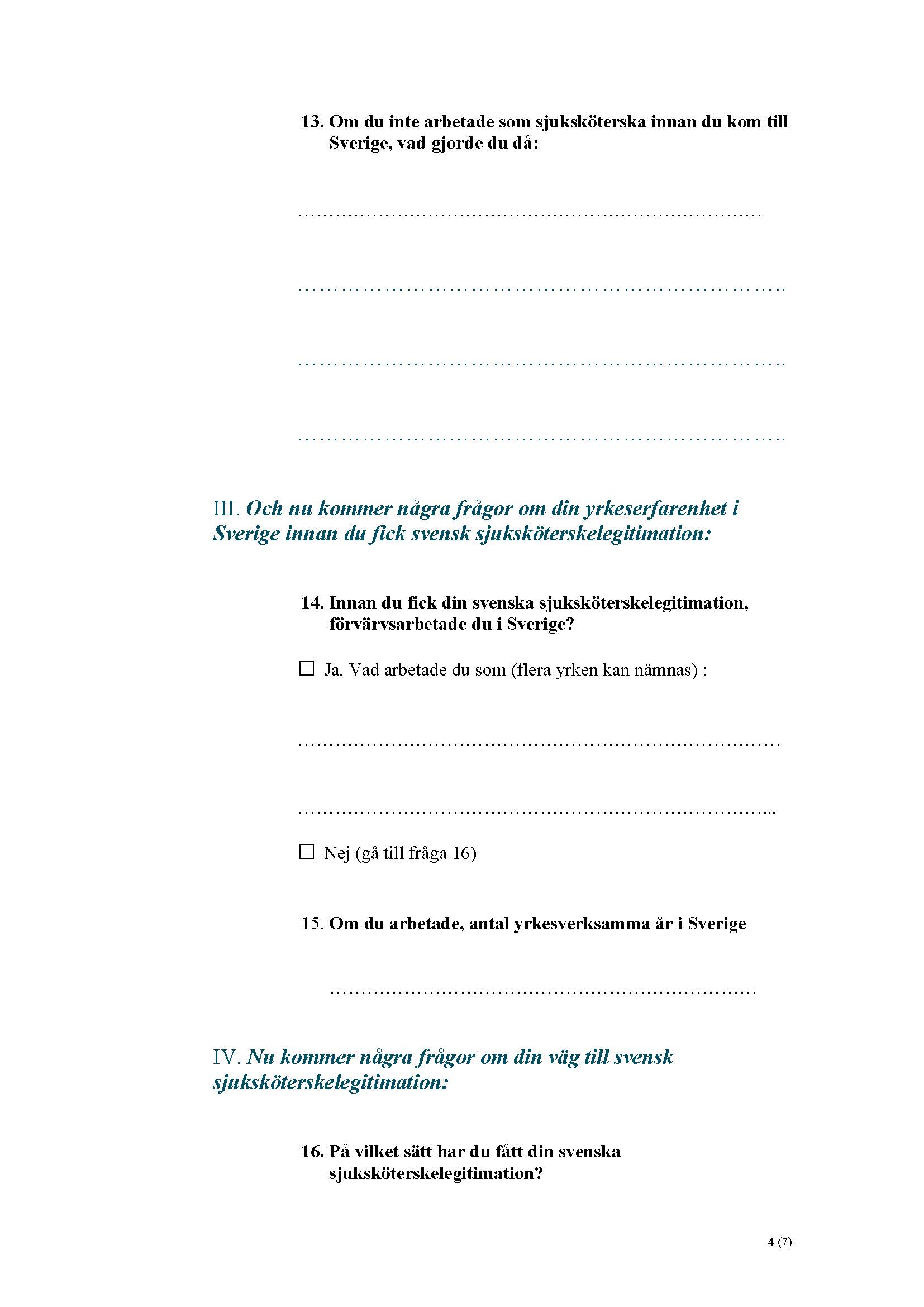

Supplement: sj-jpg-4-son-10.1177_23779608251313901 - Supplemental material for Demographic and Social Characteristics of Internationally Educated Nurses in Sweden: Descriptive Statistical Study Comparisons Between Two Different Pathways for Recertification [file sj-jpg-4-son-10.1177_23779608251313901.jpg]

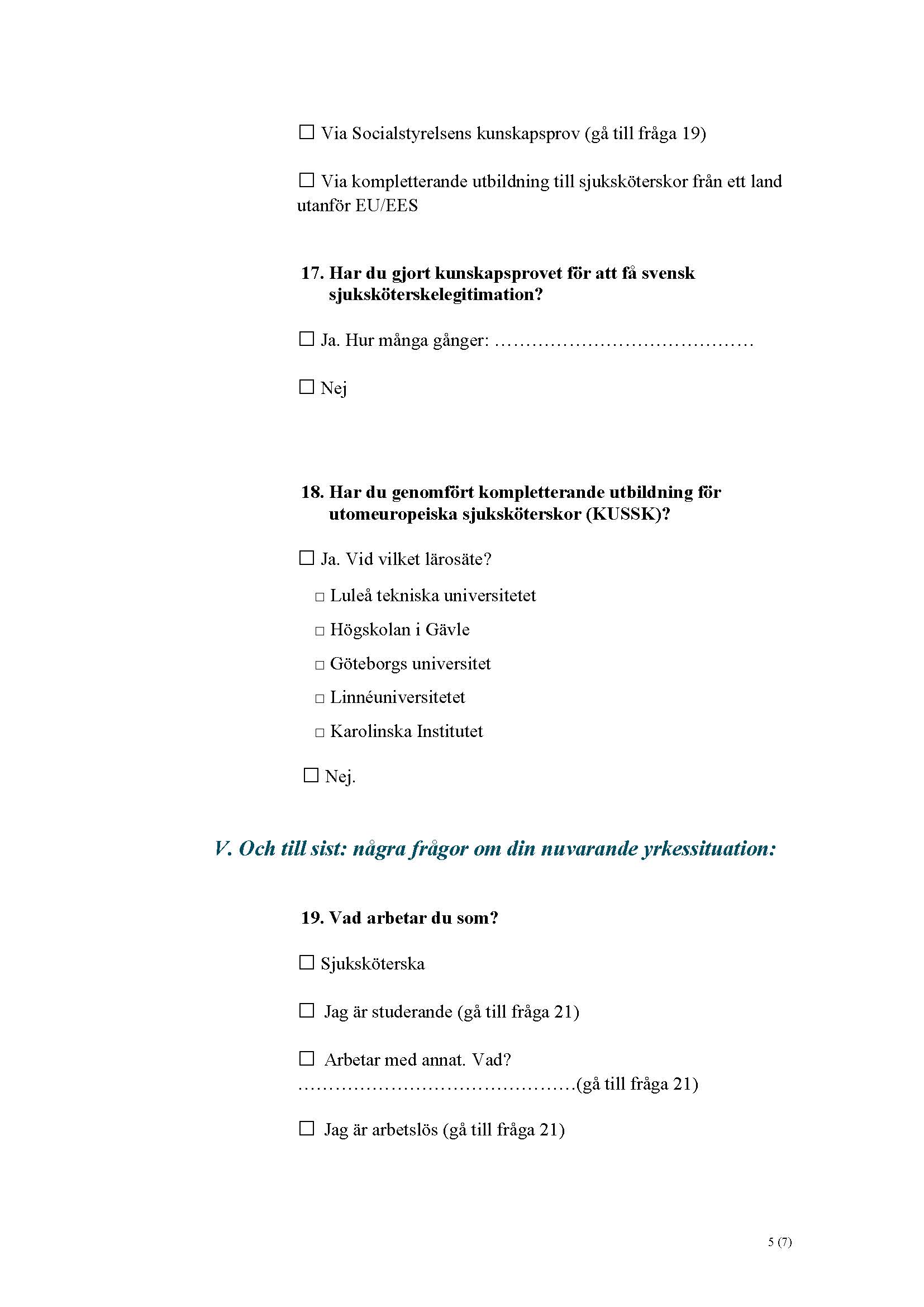

Supplement: sj-jpg-5-son-10.1177_23779608251313901 - Supplemental material for Demographic and Social Characteristics of Internationally Educated Nurses in Sweden: Descriptive Statistical Study Comparisons Between Two Different Pathways for Recertification [file sj-jpg-5-son-10.1177_23779608251313901.jpg]

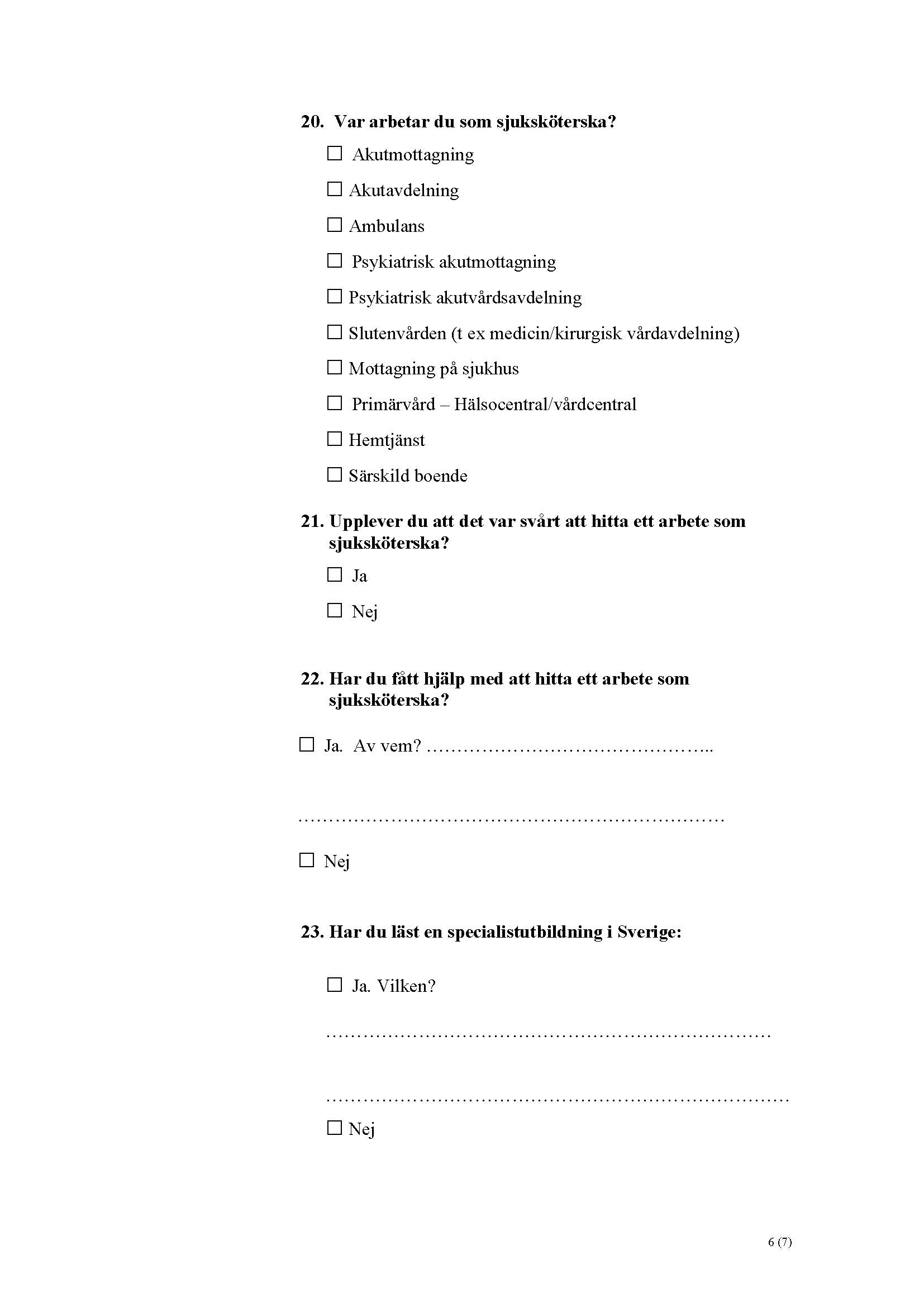

Supplement: sj-jpg-6-son-10.1177_23779608251313901 - Supplemental material for Demographic and Social Characteristics of Internationally Educated Nurses in Sweden: Descriptive Statistical Study Comparisons Between Two Different Pathways for Recertification [file sj-jpg-6-son-10.1177_23779608251313901.jpg]

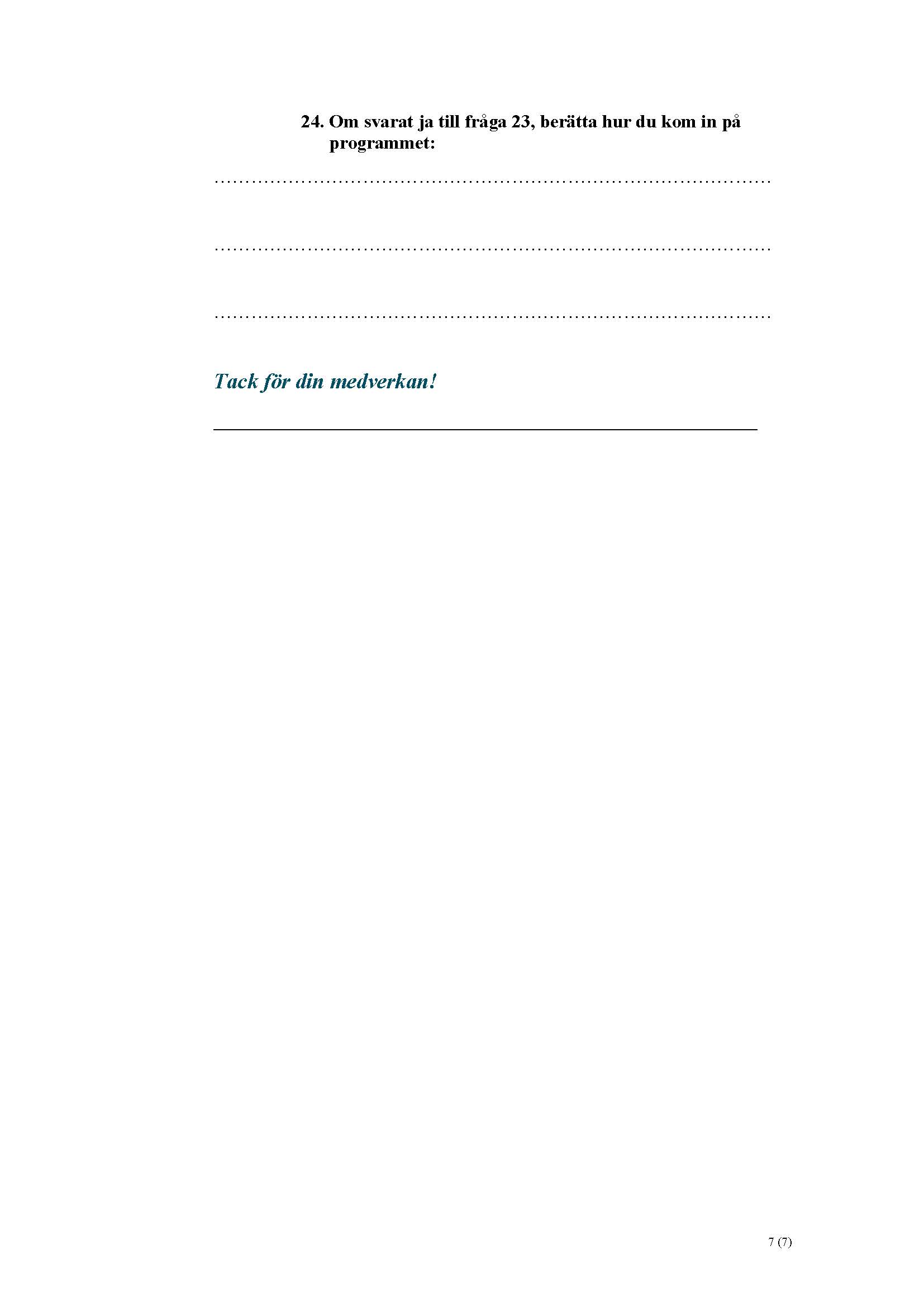

Supplement: sj-jpg-7-son-10.1177_23779608251313901 - Supplemental material for Demographic and Social Characteristics of Internationally Educated Nurses in Sweden: Descriptive Statistical Study Comparisons Between Two Different Pathways for Recertification [file sj-jpg-7-son-10.1177_23779608251313901.jpg]
